# Four-Year Outcome of Aflibercept Treatment-Naïve Patients for Neovascular Age-Related Macular Degeneration: Evidence from a Clinical Setting

**DOI:** 10.1155/2020/7465270

**Published:** 2020-03-25

**Authors:** Yanel Gayadine-Harricham, Virginie Rufin, Sandrine Law-Koune, Thi Ha Chau Tran

**Affiliations:** Ophthalmology Department, Lille Catholic Hospitals, Lille Catholic University, Lille, France

## Abstract

**Introduction:**

The objective of the study is to report 4-year treatment outcome with intravitreal Aflibercept injections for neovascular age-related macular degeneration (nAMD) as first life therapy in real-life. *Patients and Methods*. This is a prospective, monocenter, observational case series analysis. Data from treatment-naïve patients with nAMD with at least 4 years of follow-up were included in the analysis. Data including age, gender, and visual acuity measured on Early Treatment of Diabetic Retinopathy Study charts (ETDRS) and injection numbers were recorded. Spectral domain optical coherence tomography (SD-OCT) data at baseline, month 3, month 6, month 12, year 2, 3, and 4 were also recorded. Patients were treated with a modified treat and extend (T&E) regimen.

**Results:**

Of the 48 eyes with nAMD treated, only 31 eyes were available at the 4-year follow-up. The mean age was 81 ± 8 years. The VA gain was 7.3 ± 12.7 letters at 1 year 6.5 ± 12.5 letters at 2 years, VA gain 5.2 ± 17 letters at 3 years, and 6.2 ± 18.6 letters at 4 years. The reduction of central retinal thickness was 118 ± 187 *μ*m at 4 years. Complete resolution of fluid was obtained in 18/31 eyes. The total number of injections was 5.7 ± 2.0 during the first year, 2.9 ± 2.9 during the second year, 3.5 ± 3.3 during the third year, and 4.0 ± 3.4 during the fourth year. The total number of injections was 16 ± 10.6, ranging from 3 to 52 injections. Ten eyes developed macular atrophy over the 4-year period.

**Conclusion:**

The results suggest that good long-term morphological and functional outcome can be achieved using Aflibercept in clinical setting.

## 1. Introduction

Age-related macular degeneration is the major cause of blindness in the elderly [1]. Neovascular AMD (nAMD) is characterized by choroidal neovascularization (CNV), which is diagnosed by stereoscopic biomicroscopic examination of the macula, optical coherence tomography (OCT), retinal angiographies, or OCT angiography [2, 3]. Antivascular endothelial growth factor (VEGF) is the gold standard of neovascular AMD and is recommended in the international guidelines as a first-line therapy [4].

Aflibercept has been approved by the UD Food and Drug Administration in November 2011, in Europe in November 2012 [5], and was available and reimbursed in neovascular AMD in France since November 2013. Both ranibizumab and aflibercept are approved for nAMD therapy. With anti-VEGF therapy, visual gain is generally obtained at the first year, which was maintained at the second year [6, 7]. At 4 years and 5 years, visual acuity dropped to baseline level in patients of the extension study, HORIZON and CATT [8, 9], or in the real-life study [10]. After 5 years, visual acuity gradually declined thereafter in a subsequent SEVEN-UP study [11].

While the abovementioned studies used ranibizumab or bevacizumab, few data were available in long term with Aflibercept. Unlike ranibizumab and bevacizumab, aflibercept binds to VEGF-A, VEGF-B, and another protein, placental growth factor (PIGF), which is believed to play a role in progression of neovascular AMD. It has a higher affinity for VEGF and has longer half-life. Two-monthly injection of aflibercept has been shown to be safe and effective as monthly injection of ranibizumab in the treatment of nAMD in phase III of VIEW-1 and VIEW-2 studies at one year [12] and at 2 years [13]. In addition, switching to aflibercept in ranibizumab-refractory cases lead to anatomical improvement [14, 15]. Thus, we supposed that aflibercept may yield better long-term visual outcomes.

The objective of the study is to evaluate the visual and anatomical outcome at 4 years with modified T&E regimen in naïve patients treated with aflibercept and investigate the factors associated with the final vision.

## 2. Patients and Methods

### 2.1. Study Design

This is a prospective observational, consecutive case series conducted in the Ophthalmology Department of Lille Catholic Hospitals. This study was performed in accordance with the Helsinki Declaration. Institutional review board was approved by the local ethic committee, and informed consent was obtained from all patients.

### 2.2. Patients

Patients with neovascular AMD who presented to Saint Vincent hospital of Lille Catholic Hospitals from November 2013 to May 2015 and starting with Aflibercept therapy were enrolled. We have already reported the two-year results [6]. This is a consecutive study based on previous research.

Inclusion criteria included naïve patients with neovascular AMD treated with aflibercept. Exclusion criteria were (1) age ≤60 years, (2) other vitreoretinal disease, (3) intraocular surgery less than 3 months, and (4) choroidal neovascularization related to other disease than AMD.

### 2.3. Intervention and Observation Procedure

Measurement of Early Treatment Diabetic Retinopathy Study (ETDRS) best-corrected visual acuity (BCVA), intraocular pressure assessment, spectral-domain optical coherence tomography (SD-OCT), fluorescein angiography and indocyanine green angiography using a confocal laser scanning ophthalmoscope (HRA2; Heidelberg Engineering GmbH, Heidelberg, Germany) were performed at baseline. Visual acuity, adverse event monitoring, and SD-OCT were recorded at each visit. The SD-OCT-derived images had been obtained by using an eye-tracking system. Inverted images had also been routinely obtained by an enhanced depth imaging technique for the measurement of subfoveal choroidal thickness (EDI) [16]. Central retinal thickness (CRT) and macular thickness (MT) volume were computed automatically by the software (Heidelberg Eye Explorer, Heidelberg, Germany). Pigment Epithelial Detachment PED height and subfoveal choroidal thickness were manually measured. Analyses of OCT scans and variables measurements were conducted by 2 ophthalmologists (VR and YG) masked to the patient's characteristics. The presence of intraretinal fluid (IRF), subretinal fluid (SRF), hyper-reflective subretinal exudation (HSE), and disruption of ellipsoid zone (EZ) was defined as previously defined [17, 18].

### 2.4. Treatment Regimen

Participants were treated with a modified T&E regimen described previously [6] which consisted of three phases. (1) Induction phase with 3 doses of aflibercept injection (2 mg/0.05 ml) at 4-week intervals, (2) adaptation phase from week 12 to week 32, during which patients had to visit every 4 weeks and treated as needed to determine the recurrence interval, (3) after week 32, T&E was applied up to a maximum of 12-week interval during the first year and the second year. From year 2 to year 4, injection interval was extended up to 16 weeks and treatment was discontinued if there was no activity after 3 consecutive visits with injection. Once the treatment was discontinued, the monitoring protocol was monthly during the first year and then bimonthly thereafter.

If disease remained inactive through the 32-week period of observation, the patient continued the PRN dosing regimen at monthly or bimonthly visits. The retreatment criteria included recurrence of intra-/subretinal fluid or hemorrhages [19].

A treatment adherence was set up at the first visit. Explanations to patients and their relatives of importance of follow-up and treatment were given. Visits and injections were performed in the same appointments to reduce burden and fatigue. A sheet of scheduled appointments for a 6-month period was given to the patients and their family. They were requested to call in order replace a missed appointment within the week. Reimbursement for transportation was prescribed and obtained by the national healthcare system for patients if necessary. A message was sent to patients before the appointment, thanks to the reminder system set up by the hospital. When an appointment was missed, a nurse also called the patient to reschedule at the earliest a new appointment.

### 2.5. Data Collection

Data such as demographic characteristics, history of disease, history of ranibizumab treatment, and follow-up duration before and after the switch were collected from medical records and entered into an electronic file. ETDRS score, CRT, macular volume, subfoveal choroidal thickness, the maximum height of PED, the presence of intraretinal fluid (IRF), subretinal fluid (SRF), subretinal hyper reflective exudations (SHE), and disruption of the ellipsoid zone were collected. Data were collected monthly from baseline to 6 months, at 12 months, 24 months, 36 months, and 48 months. Window for data collection at the chosen annual time points was 2 months.

### 2.6. Study Outcome

The primary study outcome was change in mean VA over 48 months after initiating treatment. Secondary outcomes were change in the CRT, change in macular volume, change in subfoveal choroidal thickness, change in PED height, and mean number of injections, number of eyes with resolution of fluid, and qualitative description of OCT at different time points.

### 2.7. Statistical Analysis

Descriptive data are described as mean and 95% confidence interval or number (percentage). The statistical analysis was performed as paired comparisons between different time points using SPSS for Windows (version 20 SPSS, Chicago, IL). While we used results of survivors who completed four-year follow-up, data that included dropout patients were analyzed using last observation carried forward (LOCF) policy for the sensitivity analysis. The paired *t*-test was used for comparison between paired continuous variables. One way ANOVA was used to study the relationship between visual gain at year 4 as dependent factor and age, baseline visual acuity, baseline lesion size, and number of injections given as independent factors and four-year visual gain as dependent factor. Statistical significance was set at *P* < 0.05.

## 3. Results

### 3.1. Description of the Cohort

**Figure 1:**
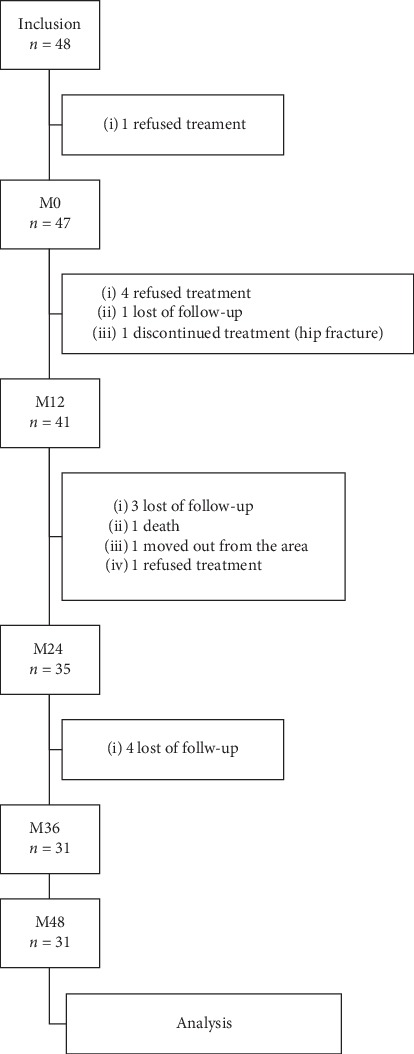
Flow chart of the cohort and survivors at year four.

Baseline clinical characteristics of the study population (survivors and dropouts) are summarized in Table 1 and Figure 1. Forty-eight eyes of 38 patients were included. In our center, the reported rate of missed/changed appointments was 11% [6]. Dropout eyes were 17/48 (35%) after the 4-year follow-up. Overall, 31 achieved the 48-month end-point. In summary, dropout patients were older at inclusion (83.6 ± 1.9 years) than patients who have completed the study (78.3 ± 1.2 years, *P*=0.013). We did not find any difference of baseline BCVA between the dropout eyes (53.1 ± 16.2 letters) and that of the eyes which achieved the 4-year follow-up (57.6 ± 16.4 letters; *P*=0.39).

### 3.2. Visual Outcome

**Figure 2:**
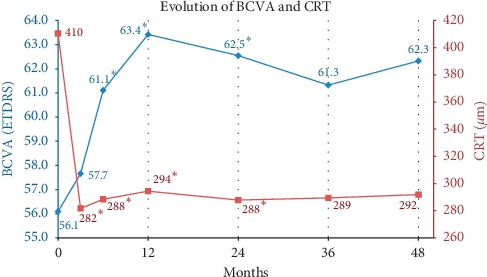
Best corrected visual acuity (BVCA -ETDRS score) and central retinal thickness (CRT) over visits. ^*∗*^*P* < 0.05.

BCVA increased from 56.1 ± 16.3 letters at baseline to 57.7 ± 20.5 letters to month 3 with a gain of +1.6 ± 11.1 letters (*P*=0.419) (Figure 2). At 6 months, visual gain was +5.0 ± 11 letters (61.1 ± 17.2 letters, *P*=0.017). At 12 months and 24 months, visual gain was still significant: +7.3 ± 12.7 letters (63.4 ± 14.8 letters, *P*=0.015) and +6.5 ± 12.5 letters (62.5 ± 19.3 letters, *P*=0.018), respectively. At 36 and 48 months, we observed letters gain of +5.2 ± 16.9 letters (61.3 ± 21 letters, *P*=0.224) and +6.2 ± 18.6 letters (62.3 ± 24.5 letters, *P*=0.162), respectively, although this difference was not statistically significant. Vision gain at 4 years was correlated with that at 6 months (*r* = 0.41, *P*=0.04) and at one year (*r* = 0.42, *P*=0.05). There was correlation between visual gain at 4 years and baseline visual acuity, baseline lesion size, and number of aflibercept injection given.

At one year, all patients have gained or maintained vision. At 2 years, 33/35 eyes (94.3%) maintained vision (loss < 15 letters), while 8/35 eyes (22.9%) earned ≥ 15 letters. At four years, (28/31) 90% maintained vision while 8/31 (26%) earned ≥ 15 letters.

The proportion of eyes with VA ≥ 70 letters, allowing driving vision, increased from baseline (14/48, 29.1%) to 16/41 (39%) at 12 months, 15/35 (43%) at 24 months, 15/31 (48%) at 36 months, and 18/31 (58%) at 48 months.

At the end of 4 years of follow-up, 8 eyes (26%) gained ≥ 15 letters ETDRS, 5 eyes (16%) gained 10–14 letters, 1 eye (3%) gained between 5 and 9 letters, 4 eyes (13%) gained between 0 and 4 letters, 10 eyes (32%) lost <15 letters, and 3 eyes (10%) lost ≥ 15 letters. Causes of loss of >15 letters was submacular hemorrhage in one case, foveal involved PED tear in one case, and foveal atrophy in one case.

### 3.3. Anatomical Response to Aflibercept

The central retinal thickness (CRT) decreased significantly from baseline (410 ± 131 *μ*m) to month 3 (282 ± 71 *μ*m, *P* < 0.001); to month 6 (288 ± 74 *μ*m, *P*=0.001); to month 12 (294 ± 75 *μ*m, *P*=0.004); to month 24 (288 ± 70 *μ*m, *P*=0.05), to month 36 (289 ± 80 *μ*m, *P*=0.463, and to month 48 (292 ± 110, *P*=0.365 *μ*m) (Figure 2).

Macular volume (MV) decreased significantly from 8.97 ± 1.21 mm^3^ at baseline to 7.97 ± 0.82 mm^3^ (*P* < 0.001) at month 3; to 8.04 ± 0.79 mm^3^ (*P* < 0.001) at month 6; to 8.03 ± 0.65 mm^3^ (*P* < 0.001) at month 12; to 7.97 ± 0.74 mm^3^ (*P* < 0.001) at month 24; to 8.07 ± 0.64 mm^3^ (*P*=0.001) at month 36, and 8.20 ± 1.28 mm^3^ (*P*=0.043) at month 48.

The PED height decreased significantly from baseline to month 3 (165 ± 97 *μ*m to 129 ± 80 *μ*m, *P*=0.014) at month 3; to 129 ± 67 *μ*m (*P*=0.02) to month 6; to 122 ± 69 *μ*m (*P*=0.01) at month 12, and to 141 ± 107 *μ*m (*P*=0.045) at month 24. After year 2, the difference in PED height from baseline was no longer significant: 140 ± 85 *μ*m (*P*=0.147) at month 36 and 147 ± 107 *μ*m (*P*=0.516) at month 48.

### 3.4. Subfoveal Choroidal Thickness

The subfoveal choroidal thickness was 192 ± 91 *μ*m at baseline; 184 ± 91 *μ*m at month 3; 185 ± 86 *μ*m at month 6; 185 ± 85 *μ*m at month 12; 184 ± 93 *μ*m at month 24; 189 ± 85 *μ*m at month 36, and 184 ± 87 *μ*m at month 48. Change in subfoveal choroidal thickness was not significant over visits.

### 3.5. Distribution of Fluid and Qualitative SD-OCT Analysis

Distribution of fluid, subretinal hyper exudation, and EZ disruption on SD-OCT was summarized in Table 2. IRF and/or SRF were present in 45/48 (94%) eyes at the beginning of the study. Complete resolution of fluid was obtained in 38/45 eyes (84%) after the induction phase, in 25/41 eyes (61%) at one year, and in 23/35 eyes (66%) at 2 years. At 48 months, 18/31 (58%) of the eyes had a complete resolution of fluid.

SHE was present in 27/48 eyes (56.2%) at baseline, in 16/45 eyes (35.6%) at month 3, in 8/41 (19.5%) at month 12, in 7/35 eyes (20%) at month 24, in 11/31 eyes (35%) at month 36, and in 10/31 eyes (32%) at month 48.

Ellipsoid zone disruption was observed in 44/48 (92%) eyes at baseline, 35/45 (78%) eyes at 3 months, in 34/41 (83%) at one year and in 31/35 (89%) at 2 years, in 27/31 eyes (87%) at month 36, and in 29/31 eyes (94%) at month 48.

Over the 4 years of follow-up, 2/48 eyes (4%) displayed a PED tear and 3/48 eyes (6%) had a subretinal hemorrhage, and 10 eyes (32%) developed a macular atrophy.

### 3.6. Frequency of anti-VEGF Intravitreal Injection

**Figure 3:**
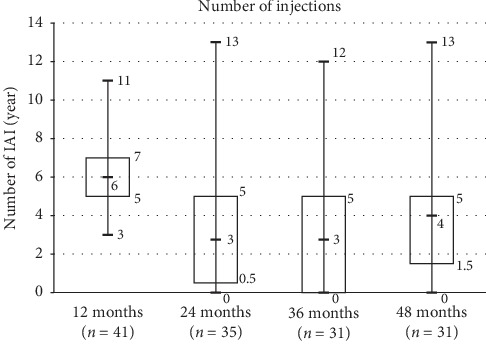
Box-plot of aflibercept injection number during the 4-year period.

Mean number of IAI was 5.7 ± 2 (median = 6, ranging from 3 to 11) during the first year, 2.9 ± 2.9 (median = 3) during the second year, 3.5 ± 3.3 (median = 3) IVT in year 3, and 4 ± 3.4 (median = 4) IVT in year 4. The average number of aflibercept injections received at the end of 4 years was 16 ± 10.6 IVT, ranging from 3 to 52 injections. There was a bimodal distribution: some patients did not require any injection after the loading phase, while 6 to 13% patients required injection every 4 weeks (Figure 3).

After the loading phase, 6 of 41 (14.6%) eyes did not have any activity through the first and second year. Among these, 2/6 were still inactive until month 48; 1/6 reactivated at the third year with maintained vision requiring 2 aflibercept injections, 1/6 died during the third year, and 2/6 were lost of follow-up. At the end of year 3, aflibercept was suspended in 7/31 (22.5%) eyes without any reactivation during year 4. At the end of year 4, suspending treatment was possible in 9/31 eyes (30%), whereas 70% others were receiving ongoing aflibercept treatment. No additional treatment was applied during this period.

**Figure 4:**
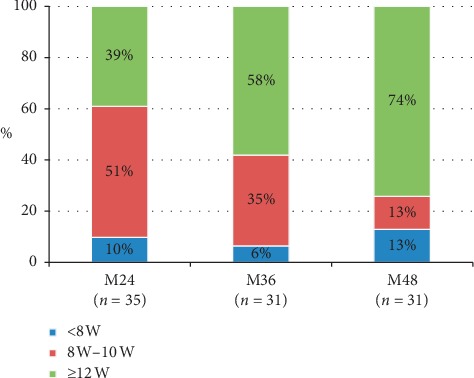
Proportion of interval treatment from year 2 to year 4.

Overall, treatment intervals of >8 weeks were found in 20/41 (48.7%) eyes during the first year and 32/35 (91.4%) during the second year. Proportion of eyes which needed interval injection ≥12 weeks increased with time, 39% at 2 years, 58% at 3 years, and 74% at 4 years (Figure 4). The maximum interval of 16 weeks was scheduled in 3 eyes and among these, 1 reactivated requiring shortened interval.

### 3.7. Adverse Events

During the 2-year study, 40/360 (11%) planned appointments had been changed or missed because of various causes (systemic disease, falls, hip fractures, stroke, and unavailability of accompanying person). Two of 7 patients who had interrupted monitoring and treatment during the first year came back to be treated during the second year. Two patients underwent cataract surgery during the 4-year follow-up. No other ocular adverse event (retinal detachment and glaucoma) occurred during the follow-up.

## 4. Discussion

In this study, we evaluated functional an anatomic response of 4-year outcome of aflibercept therapy naïve nAMD patients with a modified T&E dosing regimen in a real-life. The results showed favorable visual outcome with a mean gain of +7.3 letters at one year and of +6.5 letters at 2 years, compared with baseline. Visual gain was then described with survivors; however, the difference was not significant. The vision was maintained (losing <15 letters) in 90% eyes and most importantly, driving-vision (≥70 letters) was maintained in 58% of survivors at long term. Good anatomical response was also obtained with reduction of CRT, macular volume, and complete resolution of fluid in 58%. Two-thirds of eyes had complete resolution of fluid at 1 and 2 years and 58% of them at 4 years. Number of aflibercept injections varied widely among eyes, supporting for an individualized regimen. Additionally, we found that visual gain at 4 years was correlated with visual gain at 6 months.

Real-word clinical settings differ from clinical trial in several ways: patients were unselected and diverse; with range of comorbidity and a wider range of treatments, paradigms are implemented. It has been shown that functional stability is better in T&E compared with PRN with a 1.6 more injections but fewer clinical visits [21]. Real-world studies demonstrated that proactive dosing with aflibercept yields similar outcome to those observed in clinical trials and proactive TE-regimen is superior to PRN-regimen in clinical routine care of nAMD [22–24]. Our previous report of 2 years' results showed that aflibercept achieved similar anatomical and visual outcome using a modified T&E protocol, avoiding overtreatment in comparison with T&E real world observation registry [6].

Our real-life study also reported that accidents (fall and hip fracture), comorbidity, or relative constraints occurred 11% to 14% scheduled appointment per year, resulting in 35% missed data for the 4-year analysis in spite of effort to maximize adherence regimen. The rate of patients who dropped out was 31% in Eleftheriadou's study at 3 years [22] and 25% in Nishikawa et al.'s study at 4 years [25]. These factors can all impact on treatment outcomes [11].

The visual four-year outcome in our study is similar to Eleftheriadou's report at 3 years [22], which is to better than that of real-life studies reported by previous studies using aflibercept. We found that +6.2 letters gain was observed at 4 years and a mean number of 16 injections, though the difference was not statistically different. Eleftheriadou reported visual gain of +5.9 letters, +6.4 letters, and +6.6 letters at year 1, year 2, and year 3, respectively, with 15.6 injections at 3 years using 3 loading dose and bimonthly aflibercept injection followed by T&E regimen during year 2 and 3. Nishikawa et al. [25] investigated four-year outcome of aflibercept for nAMD and polypoidal choroidal vasculopathy, using 3 loading doses, then bimonthly aflibercept injection during the first year, and then PRN during the subsequent three years and found that visual gain obtained in the first year is gradually lost in real-world clinical practice, but vision remained above baseline level and vision was maintained in 94.5% of patients with only 15 injections. Traine et al. described in a subgroup of newly diagnosed nAMD patients with 4-year follow-up using 3 aflibercept injections of an initial loading phase following a T&E regimen that vision was stable compared to baseline (−0.7 letters, *P*=0.35) with a mean number of 7.7 injections during the first year and 4.4 injections per year from year 2 to year 4. The HORIZON study applied monthly ranibizumab injections during the first 2 years and then administered as needed in the following two years. This study showed that vision decreased. The HORIZON study showed that vision gain decreased to 2 letters and maintenance of vision was achieved in 80.4% at year four. The CATT study cohort examined the monthly bevacizumab or PRN with the switch from monthly to PRN. This study reported a loss of 3 letters at year 4 and a vision maintenance rate of 87.1%. Overall, there is a similar tendency that visual gain was no longer significant at four year, thus under different individualized regimens (3 + Q8 + PRN in Nishikawa's report, 3 + T&E in Traine's report). The 4-year data from a controlled clinical trial VIEW-1 extension, which applied a modified quarterly aflibercept injection schedule, followed by at least an every 8-week dosing through week 212, showed that vision gain maintained at 4 years with mean gain of +7.1 letters and mean number of injection 12.9 in the extension study.

Interestingly, in real-world studies, fewer numbers of injections were not associated with limited vision gain, as reported our study described in a previous study [9]. The presence of the external limiting membrane at baseline and at one year was associated with visual gain at 4 years [25]. The treatment interval extension ≥ 12 weeks was possible in half cases after year 2 and gradually increased with time up to 74% at 4 years [20] while 6% to 13% of cases need injections at ≤ 6 weeks of interval. Our modified T&E regimen with an observation period which allowed avoiding over- and undertreatment could be expected to produce good outcome with fewer injections while limiting number of visits and treatment burden on patients and caregivers.

Rate of good anatomic response defined as complete resolution of fluid was found in 58% in our study at 4 years, which was greater to that of previous reports showing persistence of fluid in 83% of eyes at 5 years using bevacizumab [8]. Rate of eyes with SHE, which represents the sign of active nAMD, was also reduced over visits. The proportion of EZ disruption and choroidal thickness reduction remained stable during the 4-year period.

The strength of our study is that (1) this is a real-world report on long-term results of aflibercept with at least 4 years of follow-up using a T&E regimen after an observation period, (2) patients were followed up and treated by the same physician ensuring the standardized personalized regimen, and (3) the set-up of the reminder system to avoid missed appointment. The limit of the study is the small number of included patients and the rate of loss of follow-up which may be responsible to a positive position leading to a better visual gain.

To conclude, in a real-world setting, treatment-naive patients with nAMD treated with aflibercept injection achieved good visual and anatomical outcome. Vision was maintained at 4 years for 90% of eyes, and 58% of eyes had VA of 20/40 or better, allowing driving-vision with an acceptable burden of the disease using an individualized regimen including observation phase and T&E regimen.

## Figures and Tables

**Table 1:** Baseline characteristics of the study population.

Number of patients (eyes)	38 (48)
Age, mean ± SD (years)	81 ± 8
Sex, *n*, male/female	
Male	12
Female	26
Bilateral disease	10
Baseline visual acuity, mean ± SD	56 ± 16
GLD, mean ± SD (mm)	2.2 ± 1.4
Surface area, mean ± SD (mm^2^)	3.8 ± 3.9
CNV type, *n* (%)	
Type 1	26 (54)
Type 2	8 [17]
Type 3	12 [20]
Polypoidal choroïdal vasculopathy	2 [4]

GLD, greatest linear diameter; CNV: choroidal neovascularization; SD: standard deviation.

**Table 2:** Qualitative OCT results over visits. SRF (sub retinal fluid), IRF (intraretinal fluid), HRD (hyper-reflective dots), SHE (subretinal hyper exudation), and EZ (ellipsoid zone) disruption.

	Inclusion *n* = 48	3 months *n* = 45	6 months *n* = 44	12 months *n* = 41	24 months *n* = 35	36 months *n* = 31	48 months *n* = 31
SRF	29 (57%)	3 (7%)	8 (18%)	5 (12%)	6 (17%)	5 (16%)	6 (19%)
IRF	25 (53%)	7 (16%)	9 (20%)	8 (20%)	10 (29%)	9 (29%)	12 (39%)
NO FLUID	3 (6%)	38 (84%)	21 (48%)	25 (61%)	23 (66%)	21 (68%)	18 (58%)
HRD	43 (91%)	36 (80%)	28 (64%)	26 (63%)	15 (43%)	22 (71%)	23 (74%)
SHE	27 (57%)	16 (36%)	14 (32%)	8 (20%)	7 (20%)	11 (35%)	10 (32%)
EZ DISRUPTION	44 (94%)	35 (78%)	30 (68%)	34 (83%)	31 (89%)	27 (87%)	29 (94%)

## Data Availability

Data are available from the authors upon reasonable request and with permission of their institution. Ethical Approval The Institutional Review Board of Lille Catholic Hospitals acknowledged the study of this cohort.

## References

[1] Weih L. M., VanNewkirk M. R., McCarty C. A., Taylor H. R. (2000). Age-specific causes of bilateral visual impairment. *Archives of Ophthalmology*.

[2] Tran T. H. C., Baglin G., Querques G. (2012). Intravitreal ranibizumab injection for choroidal neovascularization in Strümpell-Lorrain Syndrome. *Journal Français d’Ophtalmologie*.

[3] Tran T. H. C., Querques G., Forzy G., Souied E. H. (2011). Angiographic regression patterns after intravitreal ranibizumab injections for neovascular age-related macular degeneration. *Ophthalmic Surgery, Lasers, and Imaging*.

[4] Schmidt-Erfurth U., Chong V., Loewenstein A. (2014). Guidelines for the management of neovascular age-related macular degeneration by the European Society of Retina Specialists (EURETINA). *British Journal of Ophthalmology*.

[5] European Medicines Agency (2012). Eylea 40 Mg/ml solution for injection in pre-filled syringe, summary of product characteristics.

[6] Barakat A., Rufin V., Tran T. H. C. (2018). Two year outcome in treatment-naive patients with neovascular age-related macular degeneration (nAMD) using an individualized regimen of Aflibercept. *Journal Français d’Ophtalmologie*.

[7] Ohr M. (2012). Aflibercept (eylea) for age-related macular degeneration. *The Medical Letter on Drugs and Therapeutics*.

[8] Maguire M. G., Martin D. F., Ying G.-S. (2016). Five-year outcomes with anti-vascular endothelial growth factor treatment of neovascular age-related macular degeneration. *Ophthalmology*.

[9] Singer M. A., Awh C. C., Sadda S. (2012). HORIZON: an open-label extension trial of ranibizumab for choroidal neovascularization secondary to age-related macular degeneration. *Ophthalmology*.

[10] Boulanger-Scemama E., Sayag D., Ha Chau Tran T. (2016). Ranibizumab and exudative age-related macular degeneration: 5-year multicentric functional and anatomical results in real-life practice. *Journal Francais D’ophtalmologie.*.

[11] Rofagha S., Bhisitkul R. B., Boyer D. S., Sadda S. R., Zhang K. (2013). Seven-year outcomes in ranibizumab-treated patients in ANCHOR, MARINA, and HORIZON. *Ophthalmology*.

[12] Heier J. S., Brown D. M., Chong V. (2012). Intravitreal aflibercept (VEGF trap-eye) in wet age-related macular degeneration. *Ophthalmology*.

[13] Schmidt-Erfurth U., Kaiser P. K., Korobelnik J.-F. (2014). Intravitreal aflibercept injection for neovascular age-related macular degeneration. *Ophthalmology*.

[14] Tran T. H., Dumas S., Coscas F. (2017). Response to aflibercept in patients with fibrovascular pigment epithelial detachment refractory to ranibizumab in exudative age-related macular degeneration. *Journal Francais D’ophtalmologie.*.

[15] Tran T. H. C., Dumas S., Coscas F. (2017). Two-year outcome of aflibercept in patients with pigment epithelial detachment due to neovascular age-related macular degeneration (nAMD) refractory to ranibizumab. *Journal of Ophthalmology*.

[16] Spaide R. F., Koizumi H., Pozonni M. C. (2008). Enhanced depth imaging spectral-domain optical coherence tomography. *American Journal of Ophthalmology*.

[17] Gelman S. K., Freund K. B., Shah V. P., Sarraf D. (2014). The pearl necklace sign. *Retina*.

[18] Shah V. P., Shah S. A., Mrejen S., Freund K. B. (2014). Subretinal hyperreflective exudation associated with neovascular age-related macular degeneration. *Retina*.

[19] Kodjikian L., Fourmaux E., Coscas F. (2015). Traitement de la dégénérescence maculaire liée à l’âge  : avis d’experts et algorithme thérapeutique. *Journal Français d’Ophtalmologie*.

[20] Traine P. G., Pfister I. B., Zandi S., Spindler J., Garweg J. G. (2019). Long-term outcome of intravitreal aflibercept treatment for neovascular age-related macular degeneration using a “Treat-and-Extend” regimen. *Ophthalmology Retina*.

[21] Okada M., Kandasamy R., Chong E. W., McGuiness M., Guymer R. H. (2018). The treat-and-extend injection regimen versus alternate dosing strategies in age-related macular degeneration: a systematic review and meta-analysis. *American Journal of Ophthalmology*.

[22] Eleftheriadou M., Gemenetzi M., Lukic M. (2018). Three-year outcomes of aflibercept treatment for neovascular age-related macular degeneration: evidence from a clinical setting. *Ophthalmology and Therapy*.

[23] Aurell S., Sjövall K., Paul A., Morén Å., Granstam E. (2019). Better visual outcome at 1 year with antivascular endothelial growth factor treatment according to treat-and-extend compared with pro re nata in eyes with neovascular age-related macular degeneration. *Acta Ophthalmologica*.

[24] Guo M. Y., Cheng J., Etminan M., Zafari Z., Maberley D. (2019). One year effectiveness study of intravitreal aflibercept in neovascular age-related macular degeneration: a meta-analysis. *Acta Ophthalmologica*.

[25] Nishikawa K., Oishi A., Hata M. (2019). Four-year outcome of aflibercept for neovascular age-related macular degeneration and polypoidal choroidal vasculopathy. *Scientific Reports*.

